# Global, regional and national burden of male infertility in 204 countries and territories between 1990 and 2019: an analysis of global burden of disease study

**DOI:** 10.1186/s12889-023-16793-3

**Published:** 2023-11-08

**Authors:** Baoyi Huang, Zhaojun Wang, Yanxiang Kong, Mengqi Jin, Lin Ma

**Affiliations:** 1 The Reproductive Medical Center, The Seventh Affiliated Hospital, Sun Yat-sen University, No.628, Zhenyuan Rd, Shenzhen, 518107 China; 2 Department of Thoracic Surgery, The Seventh Affiliated Hospital, Sun Yat-sen University, No.628, Zhenyuan Rd, Shenzhen, 518107 China

**Keywords:** Male infertility, Global burden of disease, Years lived with disability, Years lived with disability, Socio-demographic index

## Abstract

**Background:**

Many countries and regions have experienced male fertility problems due to various influencing factors, especially in less developed countries. Unlike female infertility, male infertility receives insufficient attention. Understanding the changing patterns of male infertility in the world, different regions and different countries is crucial for assessing the global male fertility and reproductive health.

**Methods:**

We obtained data on prevalence, years of life lived with disability (YLD), age-standardized rates of prevalence (ASPR) and age-standardized YLD rate (ASYR) from the Global Burden of Disease Study 2019. We analyzed the burden of male infertility at all levels, including global, regional, national, age stratification and Socio-demographic Index (SDI).

**Results:**

In 2019, the global prevalence of male infertility was estimated to be 56,530.4 thousand (95% UI: 31,861.5–90,211.7), reflecting a substantial 76.9% increase since 1990. Furthermore, the global ASPR stood at 1,402.98 (95% UI: 792.24–2,242.45) per 100,000 population in 2019, representing a 19% increase compared to 1990. The regions with the highest ASPR and ASYR for male infertility in 2019 were Western Sub-Saharan Africa, Eastern Europe, and East Asia. Notably, the prevalence and YLD related to male infertility peaked in the 30–34 year age group worldwide. Additionally, the burden of male infertility in the High-middle SDI and Middle SDI regions exceeded the global average in terms of both ASPR and ASYR.

**Conclusion:**

The global burden of male infertility has exhibited a steady increase from 1990 to 2019, as evidenced by the rising trends in ASPR and ASYR, particularly in the High-middle and Middle SDI regions. Notably, the burden of male infertility in these regions far exceeds the global average. Additionally, since 2010, there has been a notable upward trend in the burden of male infertility in Low and Middle-low SDI regions. Given these findings, it is imperative to prioritize efforts aimed at improving male fertility and reproductive health.

**Supplementary Information:**

The online version contains supplementary material available at 10.1186/s12889-023-16793-3.

## Introduction

Millions of people worldwide experience fertility issues, with a significant prevalence observed in developing countries [1]. The decline in fertility rates gives rise to social challenges, particularly the aging population phenomenon. In a study published in 2020, researchers employed a statistical model, known as the Cohort-Component Fertility Model at Age 50 (CCF50), to project future total fertility rates across the global population. The study predicted that the world population is expected to reach its peak in 2064, while by the year 2100, a total of 183 countries are projected to have fertility rates below replacement levels [2]. Simultaneously, there is a noticeable shift in the age structure across various regions worldwide. The global forecast for 2100 indicates that the population of individuals aged 65 and above will be 1.3 times greater than the population of individuals under the age of 20 [2]. This demographic shift resulting from low fertility rates will have significant adverse implications for global development. Therefore, it is crucial to prioritize initiatives aimed at promoting fertility and addressing infertility and reproductive health issues.

Male infertility is defined as the inability to achieve conception within one year of unprotected intercourse. Studies indicate that male factors alone account for approximately 20–30% of infertility cases, while around 50% of couples experience infertility due to male factors [3, 4]. Diseases associated with male infertility primarily include obesity [5], hypogonadotropic hypogonadism, reproductive system infections [6] and systemic diseases [7]. Unhealthy lifestyles, such as smoking [8] and alcohol consumption [9], as well as environmental factors, also have detrimental effects on male reproductive function and disrupt fertility through various mechanisms [10]. Smoking and heavy drinking have been shown to negatively impact sperm quality, with alcohol consumption having a more pronounced effect on reducing sperm maturity and causing DNA damage compared to smoking [11]. Increasingly, research suggests that environmental endocrine disruptors play a significant role in the development of male infertility [12]. Among these disruptors, exposure to environmental endocrine disruptors can lead to testicular hypoplasia syndrome, which is one mechanism that affects male fertility. Furthermore, environmental endocrine disruptors may have substantial effects on reproductive function in embryos and can have long-lasting impacts on offspring through direct or epigenetic mechanisms [13].

Poor semen quality remains a prominent issue in male reproductive health. A widely used and straightforward approach to assess male fertility is the evaluation of semen quality and the detection of various semen parameters [14]. Numerous studies have been conducted to investigate and analyze trends in semen quality across different countries. Research conducted in France [15], India [16, 17], China [18], Italy [19], Uruguay [20], sub-Saharan countries [21], and other regions has consistently reported abnormally decreased male semen quality. The observed abnormal semen parameters include reduced semen count, decreased sperm motility, normal sperm morphology, decreased ejaculate volume, and prolonged sperm liquefaction time, with many studies demonstrating multiple abnormal semen parameters. Recent research has indicated a global decline in semen quality and an acceleration in sperm count reduction among males [22]. However, it is important to note that not all countries have experienced a decline in semen parameters. A Swedish study conducted in 2011 reported no decrease in semen quality or significant changes in semen parameters within their region [23]. Similarly, no declining trend in total sperm count or motility was found among men in Sydney, Australia [24]. The aforementioned studies collectively demonstrate a significant reduction in male semen quality across many countries worldwide, indicating a general global trend. Consequently, it is crucial to comprehend the disease burden of male infertility from a global, national and regional perspective.

Compared with female infertility, male infertility has not received more attention, especially in some regions. In this study, we present results from the Global Burden of Disease (GBD) 2019 and provide an assessment of current trends of diseases burden of male infertility in global, regional and national from 1990 to 2019. We hope that more attention will be paid to the global issue of male reproductive health and male infertility.

## Methods

### Overview

GBD 2019 employed standardized analytical methods to estimate epidemiological data from 1990 to 2019. This included the prevalence, disability-adjusted life years (DALYs) and years lived with disability (YLDs) of 329 diseases. Data from all eligible sources were used to determine parameters for 204 countries nested within 21 regions [25]. Compared to GBD 2017, GBD 2019 incorporated additional survey data from five countries: Italy, Nigeria, Pakistan, Philippines and Poland. This enhanced dataset provides a more comprehensive understanding of global, regional and national burden trends. The available data can be accessed at http://ghdx.healthdata.org/gbd-results-tool. Socio-demographic index (SDI) is a comprehensive indicator that provides insight into the level of development in a country or region. It is derived from a thorough evaluation of various data points, including overall fertility in women under 25 years old, the average level of education among individuals aged 15 and above, and per capita income. Ranging from 0 to 1, SDI is categorized into five groups based on the development level of countries and regions: High SDI, High-middle SDI, Middle SDI, Middle-low SDI and Low SDI [25]. We obtained the SDI data from the following website: https://ghdx.healthdata.org/record/ihme-data/gbd-2019-socio-demographic-index-sdi-1950-2019. Disability adjusted life years (DALYs) are composed of two parts: years lived with disability (YLDs) and years of life lost (YLLs). Years of life lived with disability (YLD) refers to a measure used in public health to quantify the impact of a particular health condition or disability on a person’s overall health and functioning. It represents the number of years that an individual lives in a state of reduced health, impaired functioning, or disability due to a specific disease, injury, or condition. The age-standardized prevalence rate (ASPR) is calculated by applying a standard age distribution to the observed prevalence rates of male infertility in different age groups to eliminate the effect of age structure on the rates [26]. This allows for a more accurate comparison of the prevalence of male infertility between different populations or over time, as it removes the confounding effect of age. The age-standardized YLD rate (ASYR) is calculated by applying a standard age distribution to the observed YLD rates in different age groups to eliminate the effect of age structure on the rates [26]. As there were no deaths directly attributed to male infertility, DALYs and YLDs were identical, it is important to clarify that the concept of disability addressed in this study is specific to male infertility. All estimates were accompanied by a 95% uncertainty interval (UI). According to GBD 2019, the estimates were based on age-standardized rates per 100,000 population.

### Case definition

In the GBD 2019 classification, infertility was categorized into two types: primary infertility and secondary infertility. Primary infertility refers to couples who have not achieved a live birth despite a desire for children and have been in a union for more than five years without using contraceptives. On the other hand, secondary infertility pertains to couples who desire a child and have been in a union for more than five years without using contraceptives since their last live birth. Diseases and injuries in the GBD 2019 were organized into a hierarchical structure, consisting of four levels. Level 1 represents the broadest causes of death and disability, while Level 4 comprises the most specific causes. The hierarchy encompasses three Level 1 causes, 22 Level 2 causes, 174 Level 3 causes and 301 Level 4 causes. Male infertility falls under Level 4, specifically categorized within Level 3 as urinary diseases and male infertility [27]. The International Classification of Diseases (ICD) was utilized for the classification of urinary diseases and male infertility. In the 10th edition of the ICD, the corresponding classification codes for this category include N10-N12.9, N13.6, N15, N15.1-N16.8, N20-N23.0, N25-N28.1, N29-N30.3, N30.8-N32.0, N32.3-N32.4, N34-N34.3, N36-N36.9, N39-N39.2, N41-N41.9, N44-N44.0, N45-N45.9 and N49-N49.9. In the 9th edition of the ICD, the corresponding classification codes are 588-588.9, 590-590.9, 592-593.8, 594-598.1, 598.8-599.6, 599.8, 601-602.9, 604-604.9, 608.2 and 788.0.

### Estimation methods

The estimation strategy used for male infertility in GBD 2019 is largely similar to the methods utilized in GBD 2017. Estimation is completed in three steps. First, total primary and secondary infertility in couples is estimated by quantifying the rate of infertility among married survey respondents and relating it to the overall population. Second, the proportion of primary and secondary infertility attributed to female and male factors is modeled, resulting in the estimation of four “envelopes” of infertility: male primary infertility, male secondary infertility, female primary infertility, and female secondary infertility. Third, a “causal attribution” process is carried out to assign cases within each envelope to likely underlying causes, while the remaining cases are classified as idiopathic infertility. No crosswalk was performed prior to modeling due to the lack of variability among the data sources used for estimating the infertility envelope. It was assumed that every person with infertility experiences the health state as determined by the GBD disability weights survey, with a disability weight of 0.008 for primary infertility and 0.005 for secondary infertility.

### Statistical analysis

The primary indicators employed to evaluate the burden of male infertility encompass prevalence, years lived with disability (YLD), age-standardized prevalence and age-standardized YLD from 1990 to 2019. Using the prevalence, YLD, age-standardized prevalence and age-standardized YLD data from 2019, a geographical map was generated to illustrate the spatial variations in the burden of male infertility. The percentage changes in male infertility prevalence, YLD, age standardized prevalence and age standardized YLD from 1990 to 2019 are defined as

$$\varvec{f}\left(\varvec{x}\right)=\frac{{\varvec{N}\varvec{u}\varvec{m}\varvec{b}\varvec{e}\varvec{r}}_{2019}-{\varvec{N}\varvec{u}\varvec{m}\varvec{b}\varvec{e}\varvec{r}}_{1990}}{{\varvec{N}\varvec{u}\varvec{m}\varvec{b}\varvec{e}\varvec{r}}_{1990}}\times 100\varvec{\%}$$

The relationship between the burden of male infertility and SDI was assessed based on location and year. For data analysis and visualization, we utilized R statistical software (version 4.1.2).

## Results

### Global burden of male infertility

**Fig. 1:**
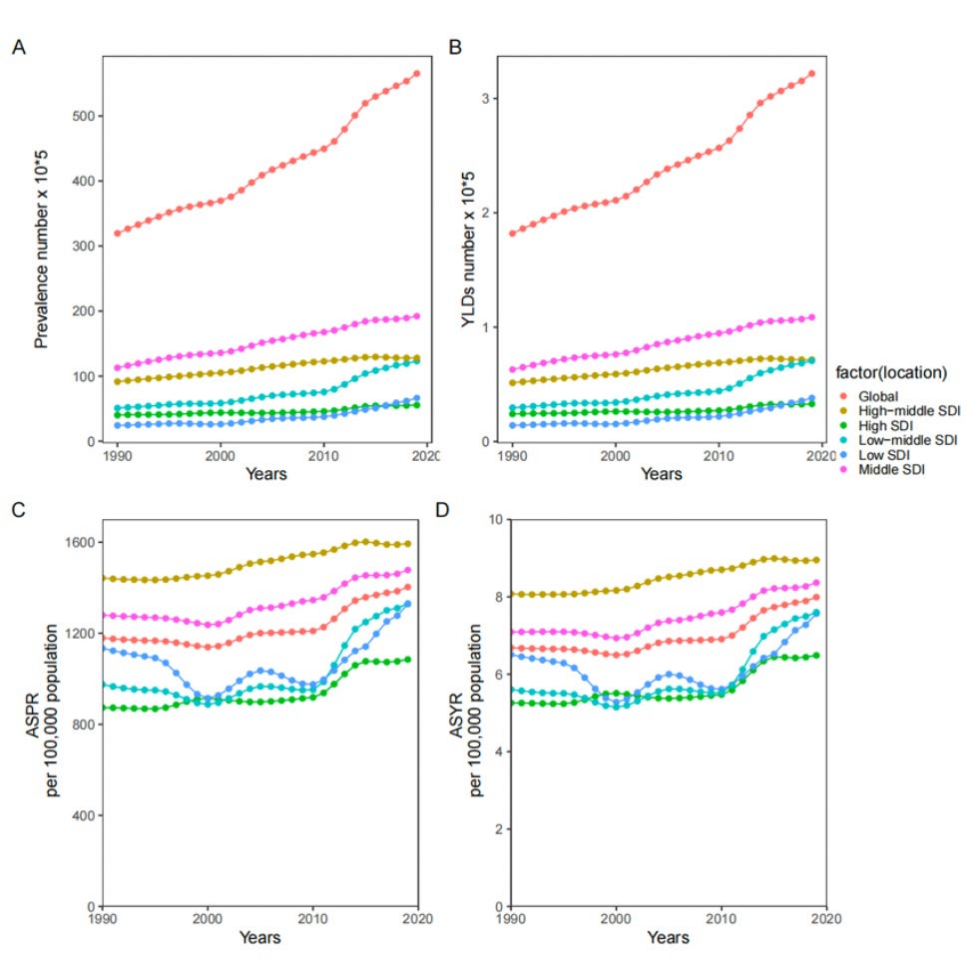
Temporal trends of male infertility prevalence number (**A**), YLD number (**B**), ASPR (**C**), and ASYR (**D**) in global, High SDI, High-middle SDI, Middle SDI, Low-middle SDI, and Low SDI from 1990 to 2019. ASPR = age-standardised prevalence rate; ASYR = age-standardised YLD rate; YLD = years lived with disability; SDI = socio-demographic index

In Fig. 1, a gradual increase in the prevalence of male infertility, represented by the YLD number, was observed globally. The ASPR and ASYR also demonstrated an upward trend between 1990 and 2019, albeit with some fluctuations along the way (Fig. 1).

To examine the global prevalence, YLD, ASPR and ASYR of male infertility and their percentage changes, a comparison was made between the data from 1990 to 2019. The prevalence number of male infertility increased by 76.9%, from 31,952 thousand (95% UI: 18,089–50,104.9) in 1990 to 56,530.4 thousand (95% UI: 31,861.5–90,211.7) in 2019. The ASPR of male infertility rose from 1,179.22 per 100,000 population (95% UI: 671.86–1,866.05) in 1990 to 1,402.98 per 100,000 population (95% UI: 792.24–2,242.45) in 2019, reflecting a 19% increase since 1990 **(**Table 1**)**.

**Table 1:** Prevalence number and age-standardized prevalence rates for male infertility in 1990 and 2019

	Prevalence number			Age-standardized prevalence rates per 100,000 population		
location	1990 No. (95%UI) *10^3^	2019 No. (95%UI) *10^3^	Percentage change	ASPR_1990	ASPR_2019	Percentage change
Global	31,952 (18,089 − 50,104.9)	56,530.4 (31,861.5–90,211.7)	76.9%	1,179.22 (671.86-1,866.05)	1,402.98 (792.24-2,242.45)	19%
Andean Latin America	59.3 (42.5–77.7)	170.9 (114.4-249.6)	188.2%	356.16 (256.51-465.56)	518.07 (346.37-761.91)	45.5%
Australasia	59.1 (33.3–97)	78.7 (44.4-128.2)	33.2%	528.81 (297.56-866.87)	567.84 (319.93-921.07)	7.4%
Caribbean	215.7 (138.3-318.6)	341.7 (197.7-555.7)	58.4%	1,255.54 (810.9-1,868.87)	1,443.49 (831.52-2,359.35)	15%
Central Asia	319.5 (188.6-502.1)	526.4 (280–900)	64.8%	947.34 (566.85-1,504.17)	1,022.01 (546.42-1,743.53)	7.9%
Central Europe	758.1 (400.4-1,285.9)	819.1 (429.6-1,412.9)	8%	1195.35 (632.67-1989.92)	1,459.9 (773.03-2,484.81)	22.1%
Central Latin America	500.9 (302.3-785.7)	1,434.6 (797.4-2,446.8)	186.4%	689.21 (419.45-1,090.08)	1,122.03 (618.48-1,899.47)	62.8%
Central Sub-Saharan Africa	285.9 (163.7-463.2)	748.9 (414-1,217.3)	161.9%	1,334.06 (754.15-2,175.75)	1,318.04 (736.64-2,163.22)	-1.2%
East Asia	12,206.5 (5983.8–20,816.1)	14,936.8 (7,597.5–25,349)	22.4%	1,751.63 (871.68-3,006.44)	1,825.6 (931-3,080.22)	4.2%
Eastern Europe	2,522.6 (1,354.2-4,101.9)	2,279.3 (1,219.1-3,745.1)	-9.6%	2,120.69 (1,141.36-3,438.8)	2,199.48 (1,192.24-3,549.31)	3.7%
Eastern Sub-Saharan Africa	648.9 (425.3-954.9)	1,775 (1,003.6-2,843.3)	173.5%	906.74 (596.77-1,332.65)	994.5 (555.7-1,636.41)	9.7%
High-income Asia Pacific	929.1 (504.9-1,577)	864.8 (464.9-1,516.4)	-6.9%	953.63 (523.7-1,607.02)	947.7 (517.47-1,581.34)	-0.6%
High-income North America	1,252.3 (707.9-2,059.7)	1,884.3 (1,076.6-3,102.9)	50.5%	783.87 (445.82-1,296.09)	1,110.14 (633.85-1,814.67)	41.6%
North Africa and Middle East	1,323.5 (941.8-1,842.1)	3793.7 (2,354-5,821.8)	186.6%	825.98 (592.46-1,131.46)	1,049.57 (652.46-1,613.22)	27.1%
Oceania	44.3 (26-68.4)	97 (56.6-151.6)	119%	1,451.57 (852.7-2,279.33)	1,446.7 (846.55-2,295.16)	-0.3%
South Asia	4,591.5 (2,610.6–7428)	12,513.3 (6,891.5–20,259.5)	172.5%	853.33 (488.12-1370.2)	1,253.87 (689.81-2,025.37)	46.9%
Southeast Asia	2,377.6 (1,340.3-3,858)	5,268.8 (2,883.2-8,511.8)	121.6%	1054.87 (590.37-1,704.01)	1,419.41 (774.61-2,301.39)	34.6%
Southern Latin America	237.9 (133.2-394.4)	346.9 (193–563)	45.8%	993.33 (557.36-1,649.3)	1,000.93 (556.69-1,633.7)	0.8%
Southern Sub-Saharan Africa	253.9 (146.9-395.4)	510.3 (279.8-820.9)	101%	1,066.01 (620.08-1,673.55)	1,173.48 (646.74-1,883.11)	10.1%
Tropical Latin America	571.6 (325.7-918.5)	1,362.4 (767.8-2,254.8)	138.3%	761.93 (437.15-1,227.84)	1,105.21 (629.43-1,839.92)	45.1%
Western Europe	1,546.7 (1,008 − 2,339)	2,079.9 (1,203.5-3,353.7)	34.5%	758.67 (494.69-1,144.05)	1,022.33 (591.01-1,667.12)	34.8%
Western Sub-Saharan Africa	1,247.2 (841.4-1,767.9)	4,697.6 (2,650.4-7,547.8)	276.7%	1,593.88 (1,081.97-2,266.57)	2,510.75 (1,403.12-3,987.3)	57.5%

Abbreviations: ASPR = Age-standardized prevalence rates; UI = uncertainty interval

In terms of YLD, the number of YLD increased from 181,947.9 (95% UI: 68,999.7–434,846.1) in 1990 to 321,829.1 (95% UI: 120,614.9–771,411.2) in 2019, indicating a 76.9% rise. Additionally, age-standardized YLD rates per 100,000 population grew from 6.68 (95% UI: 2.54–15.81) in 1990 to 7.99 (95% UI: 3.01–19.17) in 2019, representing a 19.6% increase **(**Table 2**)**.

**Table 2:** YLD number and age-standardized YLD rates for male infertility in 1990 and 2019

	YLD number			Age-standardized YLD rates per 100,000 population		
location	1990 No. (95%UI)	2019 No. (95%UI)	Percentage change	ASYR_1990	ASYR_2019	Percentage change
Global	181,947.9 (68,999.7–434,846.1)	321,829.1 (120,614.9–771,411.2)	76.90%	6.68 (2.54–15.81)	7.99 (3.01–19.17)	19.6%
Andean Latin America	342.7 (136.9-747.5)	995.2 (387.5-2,207.2)	190.4%	2.05 (0.83–4.4)	3.01 (1.18–6.66)	46.8%
Australasia	351.8 (133.6-814.8)	467.1 (178.2-1,059.7)	32.8%	3.16 (1.2–7.33)	3.38 (1.28–7.84)	7%
Caribbean	1,279.1 (486.9-2,908.3)	1,993.2 (752-4,840.6)	55.8%	7.39 (2.87–17.02)	8.41 (3.17–20.37)	13.8%
Central Asia	1,866.3 (691.1-4,498)	3,026.1 (1,100.6-7,463.6)	62.1%	5.49 (2.1–13)	5.87 (2.16–14.67)	6.9%
Central Europe	4,299.1 (1,515.4–10,807.6)	4,589.3 (1,620.1–11,010.9)	6.8%	6.81 (2.39–16.92)	8.25 (2.98–20.48)	21.1%
Central Latin America	2,886.9 (1,117.6-6,755)	8,050.1 (2,996.4–19,206.3)	178.8%	3.93 (1.52–9.15)	6.29 (2.33-15)	60.1%
Central Sub-Saharan Africa	1,612.9 (603.1-3,809.9)	4,224.2 (1,611.9–10,093)	161.9%	7.42 (2.79–17.42)	7.36 (2.78–17.34)	-0.8%
East Asia	64,666.3 (21,310.4–160,265.1)	79,907.4 (27,700.9–197,873.1)	23.6%	9.25 (3.08–22.94)	9.8 (3.37–24.36)	5.9%
Eastern Europe	14,677.2 (5,396.1–34,550.6)	13,188.6 (4,801.3–31,651.9)	-10.1%	12.39 (4.57–29.03)	12.84 (4.73–30.42)	3.6%
Eastern Sub-Saharan Africa	3,728.7 (1,475.6-8,457)	10,140.1 (3815.3–23,868.1)	171.9%	5.15 (2.06–11.93)	5.62 (2.09–13.4)	9.1%
High-income Asia Pacific	5,287 (1,960.1–11,883.9)	4,862.7 (1,788 − 10,916.2)	-8%	5.46 (2.06–12.46)	5.41 (2.04–12.25)	-0.9%
High-income North America	7,831.4 (2,890.1–18,448.9)	11,534.6 (4,317.7–25,784.5)	47.3%	4.91 (1.81–11.45)	6.81 (2.54–15.42)	38.7%
North Africa and Middle East	8,472.2 (3,435.8–18,485.3)	23,193.6 (9,054.8–52,862.9)	173.8%	5.22 (2.14–11.22)	6.43 (2.51–14.44)	23.2%
Oceania	262 (100.2-614.6)	570.8 (221.9-1,314.6)	117.9%	8.5 (3.22–19.86)	8.46 (3.3-19.23)	-0.5%
South Asia	27,517.3 (10,565.1-63695.6)	72,799.9 (26,722.8–170,871.3)	164.6%	5.07 (1.96–11.69)	7.29 (2.68–17.25)	43.8%
Southeast Asia	13,852 (5,228.2–32,977.9)	30,350.2 (11,350 − 74,346.2)	119.1%	6.11 (2.31–14.45)	8.19 (3.07–19.98)	34%
Southern Latin America	1,430.2 (536.9-3,351.9)	2,068.9 (779.8-4,742.3)	44.7%	5.96 (2.21–13.92)	5.98 (2.24–13.8)	0.3%
Southern Sub-Saharan Africa	1,486.4 (553.8-3,590.3)	2,948.7 (1,078.1-6,844.2)	98.4%	6.18 (2.31–14.59)	6.77 (2.51–15.62)	9.5%
Tropical Latin America	3,452.9 (1,313-7,984.1)	7,909 (2,998.8–17,739.1)	129.1%	4.57 (1.78–10.4)	6.43 (2.43–14.48)	40.7%
Western Europe	9611.5 (3,846.7–20,787.7)	12,659.1 (4,765.8–28,880.8)	31.7%	4.72 (1.9-10.17)	6.29 (2.35–14.27)	33.3%
Western Sub-Saharan Africa	7,034.1 (2,799.1–15,891.9)	26,350.3 (9,773.4–60,427.1)	274.6%	8.91 (3.55–19.92)	13.94 (5.13–32.3)	56.5%

Abbreviations: YLD = Years lived with disability; ASYR = Age-standardized YLD rates; UI = uncertainty interval

### Regional burden of male infertility

To compare male infertility data between 1990 and 2019, we conducted a comprehensive analysis of the prevalence, YLD, ASPR and ASYR, along with their percentage changes, across 21 regions classified by geography in the GBD study. In 2019, East Asia exhibited the highest prevalence number of male infertility, with 14,936.8 thousand cases (95% UI: 7,597.5–25,349), while Australasia had the lowest, with 78.7 thousand cases (95% UI: 44.4–128.2). Notably, only two regions, Eastern Europe and High-income Asia Pacific, experienced a decrease in the prevalence number between 1990 and 2019. Examining the ASPR of male fertility in 2019, Western Sub-Saharan Africa recorded the highest value of 2,510.75 (95% UI: 1,403.12–3,987.3), followed by Eastern Europe with 2,199.48 (95% UI: 1,192.24–3,549.31) and East Asia with 1,825.6 (95% UI: 931–3,080.22). Conversely, Andean Latin America (518.07, 95% UI: 346.37–761.91), Australasia (567.84, 95% UI: 319.93–921.07) and High-income Asia Pacific (947.7, 95% UI: 517.47–1,581.34) exhibited the lowest ASPR values. Regarding the percentage change in ASPR from 1990 to 2019, Central Latin America observed the highest percent change at 62.8%, followed by Western Sub-Saharan Africa at 57.5%, and South Asia at 46.9%. Interestingly, three regions experienced a decrease in ASPR percentage change in 2019 (Central Sub-Saharan Africa, High-income Asia Pacific and Oceania), while ASPR increased in eighteen regions. Notably, two regions, Western Sub-Saharan Africa and Central Latin America, witnessed an increase in ASPR by over 50% **(**Table 1**).**

Similarly, in 2019, East Asia recorded the highest YLD number for male infertility, with 79,907.4 (95% UI: 27,700.9–197,873.1), followed by South Asia with 72,799.9 (95% UI: 26,722.8–170,871.3) and Southeast Asia with 30,350.2 (95% UI: 11,350–74,346.2). Notably, Western Sub-Saharan Africa exhibited the largest percentage change in YLD at 274.6%. On the other hand, only Eastern Europe (-10.1%) and High-income Asia Pacific (-8%) experienced a decrease in the percentage change, while the remaining nineteen regions showed an increase. Furthermore, in 2019, Western Sub-Saharan Africa had the highest ASYR per 100,000 population, with a value of 13.94 (95% UI: 5.13–32.3), followed by Eastern Europe with 12.84 (95% UI: 4.73–30.42) and East Asia with 9.8 (95% UI: 3.37–24.36). Conversely, Andean Latin America was the region with the lowest ASYR, with a value of 3.01 (95% UI: 1.18–6.66). Between 1990 and 2019, an increasing trend in ASYR was observed in eighteen regions, while only three regions, High-income Asia Pacific, Central Sub-Saharan Africa and Oceania, experienced a decrease in ASYR **(**Table 2**)**.

### National burden of male infertility

**Fig. 2:**
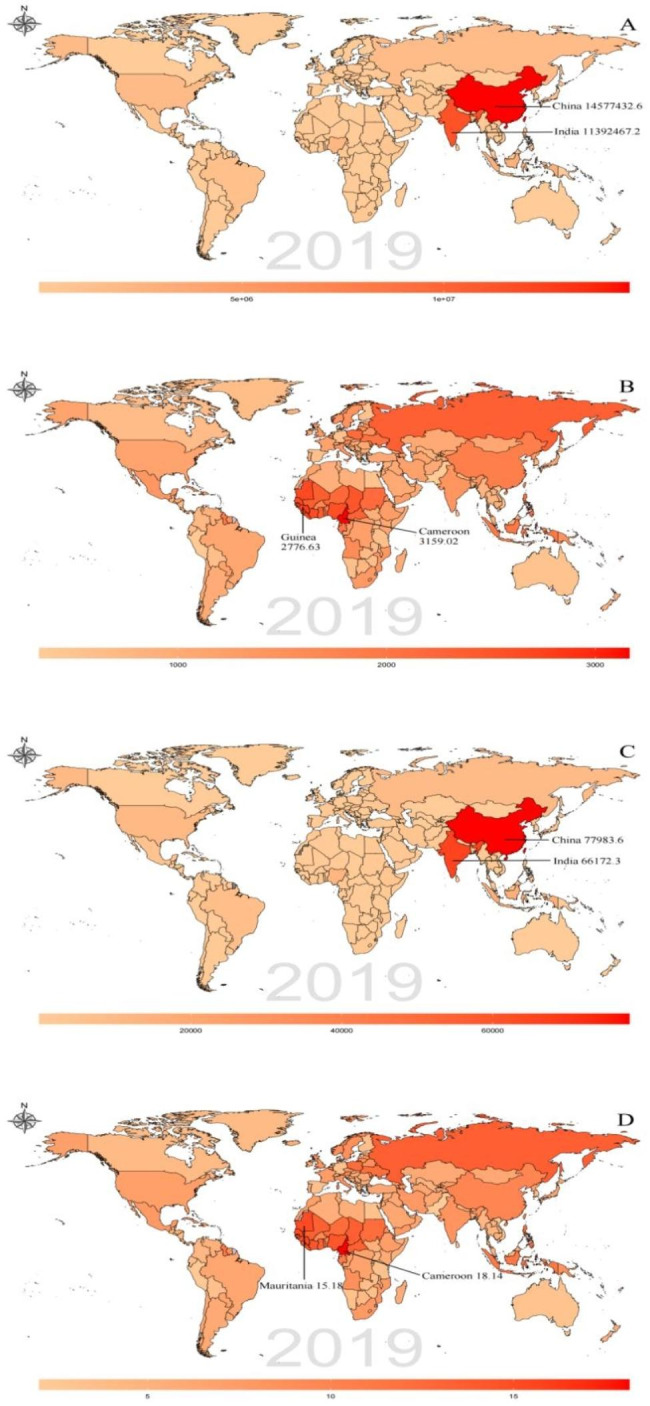
Global distribution of male infertility burden in terms of prevalence number (**A**), ASPR (**B**), YLD number (**C**) and ASYR (**D**) in 2019. ASPR = age-standardized prevalence rate; ASYR = age-standardized YLD rate; YLD = years lived with disability

In Fig. 1, a gradual increase in the prevalence of male infertility, represented by the YLD number, In addition to regional analysis, this study also compared the data among 204 countries and territories in 2019. Among these countries and territories included in the GBD study, China exhibited the highest prevalence number of male infertility, with 14,577,432.6 (7,416,528.3–24,752,355.6), followed by India with 11,392,467.2 (6,258,342–184,672,22) and Indonesia with 2,873,683.7 (1,563,954.3–4,590,378.2) **(**Fig. 2A **and additional file 1)**. Similarly, the ranking for YLD number mirrored the prevalence ranking, with China being the highest at 77,983.6 (27,050.6–193,046.6), followed by India with 66,172.3 (24,375.1–156,501.1) and Indonesia with 16,430.3 (6,021.4–39,065.8). Notably, Tokelau had the lowest YLD ranking, with a value of 0.1 (0-0.1) **(**Fig. 2 C **and additional file 3)**. Examining the ASPR, the top five countries with the highest ASPR values were Cameroon with 3,159.02 (1,828.63–5,022.17), Guinea with 2,776.63 (1,556.16–4,588.61), Senegal with 2,685.8 (1,570.06–4,262.13), Liberia with 2,684.92 (1,473.85–4,402.04) and Mauritania with 2,658.04 (1,536.9–4,303.08) **(**Fig. 2B **and additional file 2)**. Similarly, the top five countries with the highest ASYR values were Cameroon with 18.14 (6.73–42.12), Mauritania with 15.18 (5.77–35.62), Senegal with 15.16 (5.62–35.24), Guinea with 15.13 (5.35–35.78) and Liberia with 14.61 (5.25–33.95). Conversely, Pakistan had the lowest ASYR ranking, with a value of 2.06 (0.7–5.01) **(**Fig. 2D **and Additional file 4)**.

### Age pattern

**Fig. 3:**
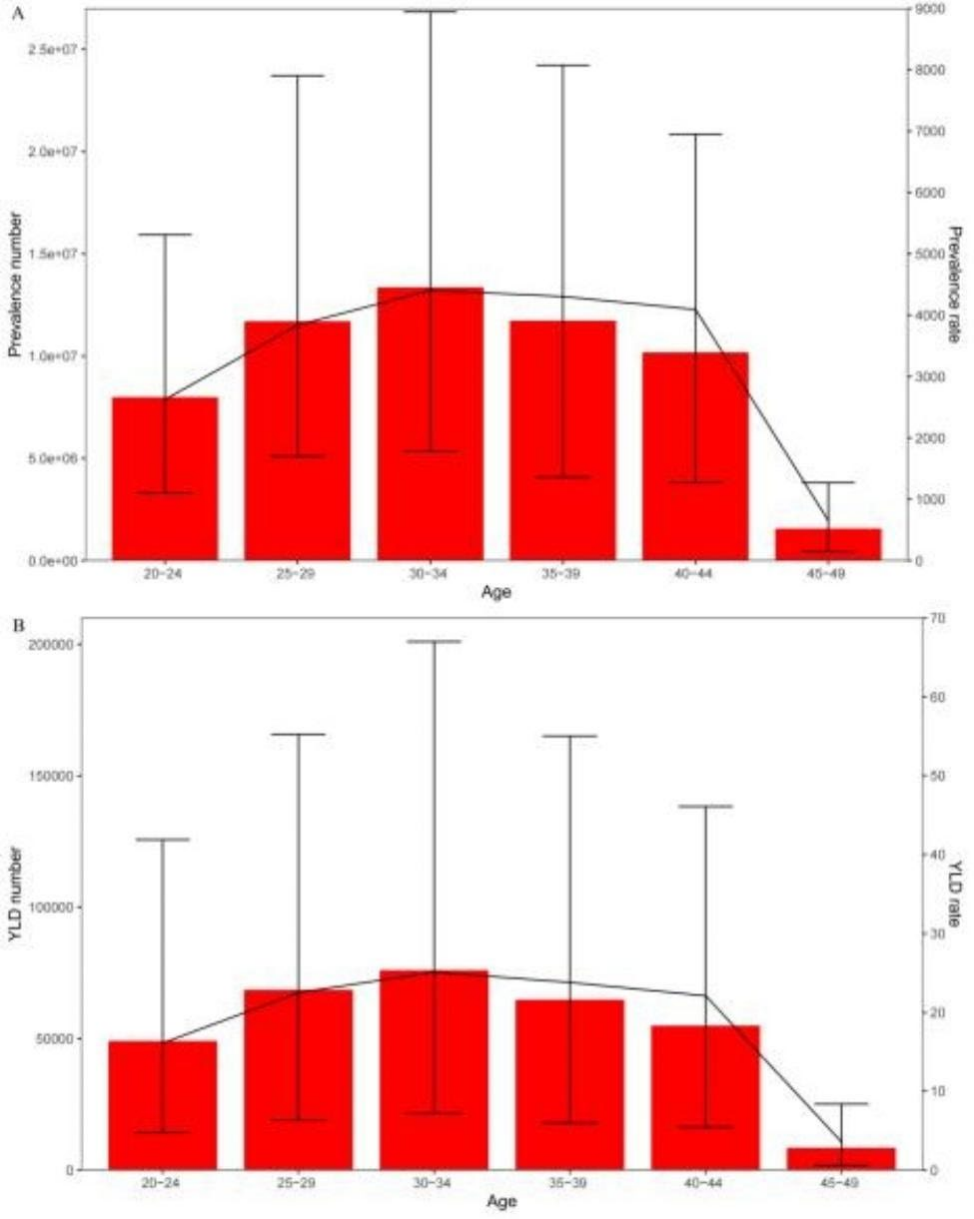
Age-specific numbers and rates of prevalence and YLD of male infertility in 2019. (**A**) prevalence. (**B**) YLD. YLD = years lived with disability

When examining the prevalence and YLD of male infertility across different age groups through age stratification, we observed that the global prevalence number, prevalence rate, YLD number and YLD rate of male infertility reached their peak in the 30–34 year age group in 2019 (Fig. 3). In the prevalence age-stratified analysis, the number and rate of prevalence were comparable between the 25–29 and 35–39 year age groups **(**Fig. 3A**)**. However, in the YLD age-stratified study, the number and rate of YLD were slightly higher in the 25–29 year age group compared to the 35–39 year age group **(**Fig. 3B**)**. Notably, both the prevalence and YLD numbers of male infertility exhibited a significant reduction in the 40–45 year age group when compared to other age groups **(**Fig. 3**)**.

### Burden of male infertility by SDI

Figure 1 provides an overview of the temporal trends in the prevalence number, YLD number, ASPR and ASYR of male infertility from 1990 to 2019, categorized by different levels of SDI, including High SDI, High-middle SDI, Middle SDI, Low-middle SDI and Low SDI.

Over the study period, both the prevalence and YLD numbers in Middle SDI regions exhibited a gradual increase. The High-middle SDI regions closely followed the Middle SDI regions, with prevalence and YLD numbers consistently remaining at high levels. In contrast, the prevalence and YLD in High SDI regions remained relatively low. Notably, Middle SDI and High-middle SDI regions had the highest prevalence and YLD numbers, while High SDI and Low SDI regions had the lowest **(**Fig. 1A and B**)**. In terms of ASPR and ASYR, the burden of male infertility in High-middle SDI and Middle SDI regions surpassed the global average. Conversely, the ASPR and ASYR of male infertility in High SDI, Low-middle SDI and Low SDI regions were lower than the global levels. However, it is important to highlight that the ASPR and ASYR of Low-middle SDI and Low SDI regions exhibited a significant increase after 2010. Furthermore, the ASPR and ASYR showed an increasing trend in all five SDI regions between 1990 and 2019. Among these regions, High-middle SDI had the highest values, while High SDI had the lowest. However, the patterns of change differed among the regions, with High-middle SDI displaying a continuous rise and Middle SDI, Low-middle SDI and Low SDI regions demonstrating a W-shaped pattern of change **(**Fig. 1C and D**)**.

**Fig. 4:**
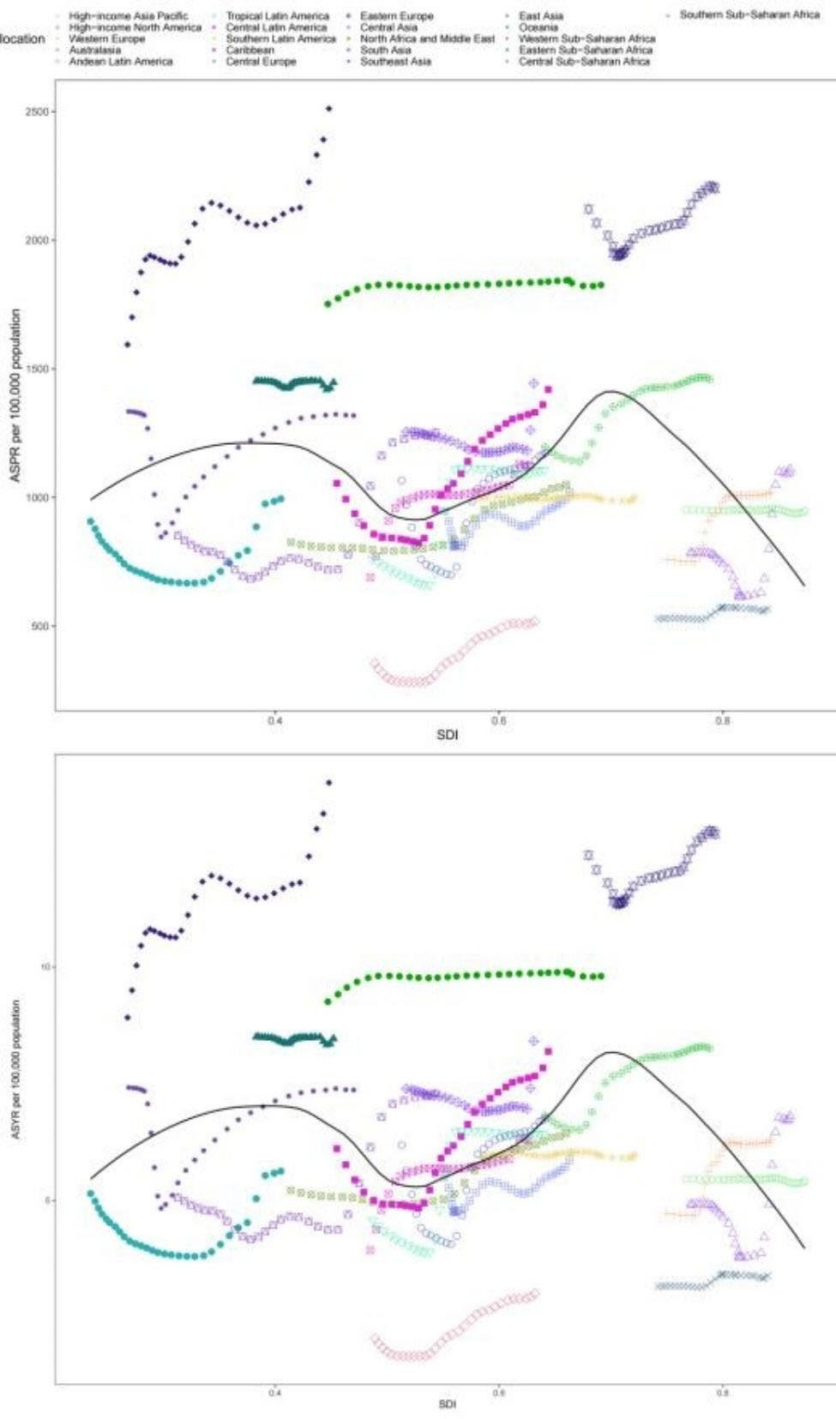
Age-standardised prevalence rates (ASPR) and age-standardised YLD rates (ASYR) for male infertility in 21 GBD regions by Socio-demographic Index, 1990–2019. Expected values based on Socio-demographic Index and disease rates in all locations are shown as the black line. YLD = years lived with disability

In our study, we examined the correlation between SDI and the corresponding ASPR and ASYR of male infertility in 21 regions covered by the GBD study, spanning the period from 1990 to 2019. Our analysis revealed an M-shaped relationship between SDI and the ASPR and ASYR of male infertility. At the regional level, the expected values displayed two peaks at SDI values of 0.4 and 0.7. Additionally, when the SDI value was 0.5, a slight decline was observed, followed by a subsequent increase. However, for SDI values greater than 0.7, the ASPR exhibited a substantial decreasing trend. During the study period, the ASPR and ASYR in Western Sub-Saharan Africa, East Asia, Eastern Europe, Oceania and the Caribbean were higher than the expected values based on their respective SDI levels. On the other hand, the ASPR and ASYR in Eastern Sub-Saharan Africa, North Africa and the Middle East, Southern Latin America, Andean Latin America and Australasia were lower than the expected values. Notably, in the early years, a lower burden of ASPR was observed in South Asia, Southeast Asia, Southern Sub-Saharan Africa, Western Europe, High-income Asia Pacific and High-income North America. However, it is important to highlight that in the latter years, the burden of ASPR increased in these regions. Overall, our findings suggest a complex relationship between SDI and the burden of male infertility, characterized by regional variations and changes over time **(**Fig. 4A and B**)**.

## Discussion

Male infertility represents a significant burden globally, with implications for individuals and societies across the world. This study aimed to investigate the global, regional, and national burden of male infertility across 204 countries and territories from 1990 to 2019. Our findings indicate a substantial increase in the prevalence of male infertility since 1990. The burden was particularly pronounced in certain regions, such as Western Sub-Saharan Africa, Eastern Europe, and East Asia, which exhibited the highest prevalence rates. Notably, the 30–34 years age group showed the highest prevalence and YLD on a global scale. Furthermore, the burden of male infertility in High-middle SDI and Middle SDI regions exceeded the global average, emphasizing the urgent need for targeted interventions and public health strategies to address this growing issue.

In this study, we assessed the prevalence of male infertility globally. According to the results, more than 56 million men suffered infertility up to 2019 globally. The prevalence of male infertility has increased from 1990 to 2019, with a growth rate of 76.9% compared to 1990. This increase is significant, especially when considering that the world population has only increased by 46% during the same time period. Our findings clearly demonstrate of the global burden of male infertility, as measured by prevalence, ASPR and ASYR, increased progressively between 1990 and 2019. Regarding the issue of whether male fertility has decreased, most studies currently show a significant decrease in male fertility, but a small number of studies hold reservations [28]. In 2019, a global infertility burden study from 1990 to 2017 in 195 countries showed that the age-standardized prevalence of infertility increased every year in both male and female, with male infertility increasing at a rate of 0.291% per year [25]. The most common manifestation of male infertility was a decrease in semen quality in men. As early as 1992, a study on male semen quality statistically analyzed all literature on semen quality in men without a history of infertility between 1938 and 1991, and found that the mean sperm count and semen volume in men decreased significantly, indicating that male semen quality had been abnormal during the 50 years from 1938 to 1991, reflecting that overall male fertility had gradually decreased in the 1990s [29]. In recent years, studies on male semen in several countries have shown a gradual decrease in male semen quality, mainly reflected in a decrease in sperm concentration and an increase in the rate of abnormal sperm morphology [15, 30–32]. In 2023, a study has found that semen quality declines and sperm count continues to decline in men worldwide, which was getting more severe after 2000 [22]. The phenomenon of decreased semen quality in men worldwide explained the general trend of increasing prevalence of male infertility year by year. Besides, a systematic literature review was conducted on whether infertility and its treatment affect the sexual life of couples. It was found that infertility has a negative impact on the sexual life of infertile couples, with the proportion of male sexual dysfunction ranging from 48 to 58% [33]. The global prevalence of male infertility is increasing, with an increasing number of affected populations and a significant threat to male reproductive health worldwide.

About half of infertility couples have male factors [34], including common male factors such as abnormal semen quality, non liquefaction of semen, erectile dysfunction, ejaculation disorders, and retrograde ejaculation. Some males achieve satisfactory outcomes after correcting infertility factors after receiving treatment, while some male patients are still unable to conceive their partners after receiving treatment, like antioxidants, or inositol [35]. These infertile couples have to receive human assisted reproductive technology (ART) to assist in pregnancy. A systematic review study found that in terms of cognitive ability to accept infertility, men with higher levels of cognition showed higher levels of anxiety and lower treatment adherence intentions [36]. The psychological disorders of infertile men are one of the factors that affect patients’ compliance with reproductive therapy and treatment effectiveness. At present, assisted reproductive technology has been widely applied globally, and with the progress of medical technology, a series of derivative technologies related to assisted reproductive technology have been created, such as Intracytoplasmic sperm injection (ICSI), embryo freezing technology, etc. However, there are still many doubts about the health issues of offspring with assisted reproductive technology, especially after birth, such as the long-term effects of embryo freezing on congenital malformations [36] and mental health in children, such as intelligence, cognitive ability, and mental health [37]. A study on newborns born from frozen embryos and their mid to long-term follow-up outcomes suggests that frozen embryos may have an impact on neonatal birth weight, increasing the incidence of macrosomia and LGA (greater than gestational age) newborns. However, there is no significant difference in the incidence of congenital malformations and neurodevelopmental abnormalities in mid to long-term studies. Additionally, the birth of frozen embryos may be associated with a higher prevalence of infectious diseases, respiratory diseases, and nervous system diseases [38]. At the same time, the impact of severe male infertility on the reproductive health of offspring is also a focus of attention. Research has found that severe male factor infertility and the use of ICSI can both lead to a small increase in the risk of intellectual disability and autism in offspring. Research has found that there are sperm abnormalities in adult males who are pregnant with ICSI [39], and due to the small number of children born under ICSI treatment, the accuracy of the data is still questionable. Recently, artificial intelligence (AI) has also been applied to the assessment of male infertility. By assessing lifestyle and environmental factors, AI can be used to predict male fertility and semen quality with good predictability [40]. At the same time, artificial intelligence uses machine learning to better select the best sperm for in vitro fertilization, guide nutritional supplementation, and improve male infertility.

Infertility is a major reproductive health problem worldwide and is estimated to affect 8–12% of couples of reproductive age [4]. Infertility appears almost exclusively in couples of reproductive age, except for some elderly couples who accidentally lose their only child. The occurrence of infertility may involve male, female and couple factors, but due to the influence of social and traditional concepts, people always root infertility in women. In a retrospective analysis in 2017, it was found that the prevalence of infertility in India was as high as 45%, influenced by local traditional culture and perceptions of infertility, and many Indians believed that infertility was a curse of God, while men were not considered responsible for the matter of inability to conceive [41]. In this study, we found that the highest prevalence number was observed in China, followed by India. The higher prevalence in China and India could be attributed to their large populations and demographic factors such as delayed childbearing, urbanization and changes in lifestyle. These factors may contribute to increased infertility rates due to factors like pollution, occupational hazards, and access to reproductive healthcare services.

At region level, we found that Western Sub-Saharan Africa, Eastern Europe and East Asia had the highest age-standardized prevalence rate of male fertility, especially Western Sub-Saharan Africa, with total and growth ASPR well ahead of other regions in 2019. The top five ASPR and ASYR countries rankings were not exactly the same but similar, mostly in Western African countries. It suggested that there was a huge burden of infertility among men in these countries, especially in Western Africa. In 2015, a study of the prevalence of male infertility in different regions of the world showed that Africa and Central and Eastern Europe had the highest infertility rates, especially the “African Infertility Belt” The prevalence of female infertility in this region was also very high, with male factor infertility accounting for about 43% of the responsibility. It may be due to backward medical care, sexually transmitted diseases (STDs) and genital tract infections in Africa, which affect male fertility [42].

Infertility stands apart from other diseases due to its unique characteristic as a demand-driven condition. Unlike many illnesses that require medical attention irrespective of personal choices, infertility is only deemed significant when a couple desires to conceive but faces difficulties. This poses challenges in collecting accurate statistical data on its prevalence. Individuals who are subfertile but lack a strong desire for biological parenthood may not actively seek medical interventions, leading to an underestimation of infertility’s true prevalence. The data from developed countries such as North America, Europe and Australia has been considered the most comprehensive and accurate, as the data from these regions was derived from the National Health Statistics Report (NHSR), the Australian Institute for Health and Welfare (AIHW), and the European Association of Urology (EAU) guidelines for male infertility, with the most accurate reporting data from these organizations [42]. To obtain a more accurate understanding of infertility’s prevalence, a comprehensive approach considering medical data, sociocultural factors, and reproductive intentions is crucial. Longitudinal studies, population surveys and data triangulation from multiple sources can enhance knowledge about the prevalence and burden of infertility, addressing the challenges posed by its demand-driven nature and enabling better support for individuals, couples, and societies facing infertility-related issues.

Our study found a non-linear relationship exists between SDI and the corresponding ASPR and ASYR of male infertility. We found that the expected values of ASPR and ASYR for male infertility reached their peak at SDI values of 0.4 and 0.7, and were at a trough at SDI values of 0.5. Besides, a negative correlation is observed when SDI exceeds 0.7. As SDI increases from lower levels, the burden of male infertility tends to rise. This is evidenced by the increasing ASPR and ASYR values until an SDI value of 0.4. Regions with moderate levels of development experience the highest burden of male infertility, possibly due to factors such as changing lifestyles, environmental exposures, and increased healthcare access, which can contribute to fertility issues. However, as SDI surpasses 0.7, we noted a negative correlation between SDI and the burden of male infertility. Highly developed regions experienced lower burdens, reflected by decreasing ASPR and ASYR values. This could be explained by factors such as advanced healthcare facilities, better access to fertility treatments, higher socioeconomic conditions, and improved overall health and well-being, leading to decreased fertility issues in these regions. These findings highlight the complex interplay between socio-demographic factors and the burden of male infertility, emphasizing the need for targeted interventions and policies tailored to different levels of development.

In some developing countries, severe pollution levels and endocrine disruptors prevalent in the environment contribute significantly to the decline in fertility parameters [17]. A Chinese study found that sperm density declined faster in students than in non-students. These results suggested that sedentary behavior, lack of sleep, high psychosocial stress and lifestyle of college students with prolonged smartphone and Internet use adversely affect semen quality [18]. A study analyzed causes of infertility in sub-Saharan Africa mentioned that the main underlying cause of high infertility levels in this region appeared to be infectious diseases, especially Neisseria gonorrhoeae infection, manifesting as obstructive azoospermia. Other infections, such as syphilis, tuberculosis may also be important causes [6]. In 2015, a study addressed six demographic characteristics of infertility worldwide, including in Africa, with a very high prevalence of infertility, while a paradoxical point is that high infertility rates coexist with high fertility rates. In high fertility areas such as sub-Saharan Africa, lack of infertility prevention and treatment services was considered a form of population control [1]. The lack of infertility diagnosis and treatment techniques and institutions as well as errors in the concept of infertility treatment may be one of the reasons for the high prevalence of male infertility in poor areas such as sub-Saharan Africa.

Theoretically, age is also an important factor affecting prevalence. The occurrence of infertility is usually mixed with both male and female factors, so there is high relation between men and women age [43, 44] and that the delayed childbearing desire age is a major and well documented impact in women that can induce some interference in this analysis as the concept of male infertility is sometimes doubtfull and accompanied by female factors. In this study, the age group of 30–34 year age showed the highest prevalence of infertility and the heaviest fertility burden. However, men in other age groups with high fertility needs also showed a higher burden, suggesting that the burden of infertility coexists with high fertility needs. Several factors could contribute to this observation. Firstly, biological factors such as age-related decline in sperm quality and quantity could play a role. Additionally, lifestyle factors including delayed childbearing, increased stress levels, sedentary behavior and unhealthy dietary habits may contribute to the higher prevalence of infertility in this age group. Moreover, socioeconomic factors such as career aspirations, financial constraints and limited access to reproductive healthcare services might also influence fertility outcomes.

Our study on the global burden of male infertility from 1990 to 2019 has certain limitations. Data sparsity, particularly in underdeveloped countries, affects disease estimation at the population level. Cultural differences and patriarchal societies influence people’s understanding of male infertility and may impact the accuracy of statistical data collection. Additionally, misconceptions about male infertility can affect patient acceptance of infertility treatment. Despite these limitations, our study represents the most comprehensive epidemiological investigation to date. Future research should address these limitations by improving data collection methods and promoting awareness and understanding of male infertility in different cultural contexts to provide more accurate assessments of the disease burden and facilitate effective interventions for those affected.

## Conclusion

The escalating prevalence of male infertility has emerged as a pervasive global trend, with multiple regions experiencing a notable increase in its prevalence. This upward trajectory underscores the ongoing erosion of male reproductive health and fertility on a significant scale worldwide. Particularly concerning is the situation in Western Sub-Saharan Africa, where the burden of male infertility has exhibited the most severe rise. Interestingly, while the burden of male infertility did not show a direct association with SDI in this study, it is noteworthy that male reproductive burden in high SDI regions remained relatively low. Nonetheless, the current status of male infertility poses a substantial threat to reproductive health and necessitates heightened attention from researchers.

### Electronic supplementary material

Below is the link to the electronic supplementary material.

Supplementary Material 1

Supplementary Material 2

Supplementary Material 3

Supplementary Material 4

## Data Availability

The datasets analysed during the current study are available in the The Global Burden of Disease (GBD) 2019 repository, http://ghdx.healthdata.org/gbd-results-tool.
